# Key role of activated platelets in the enhanced adhesion of circulating leucocyte-platelet aggregates to the dysfunctional endothelium in early-stage COPD

**DOI:** 10.3389/fimmu.2024.1441637

**Published:** 2024-08-19

**Authors:** Patrice Marques, Irene Bocigas, Elena Domingo, Vera Francisco, Julia Tarraso, Yolanda Garcia-Sanjuan, Esteban J. Morcillo, Laura Piqueras, Jaime Signes-Costa, Cruz González, Maria-Jesus Sanz

**Affiliations:** ^1^ Department of Pharmacology, Faculty of Medicine and Odontology, University of Valencia, Valencia, Spain; ^2^ Institute of Health Research INCLIVA, University Clinic Hospital of Valencia, Valencia, Spain; ^3^ CIBEREHD-Spanish Biomedical Research Centre in Hepatic and Digestive Diseases, Carlos III Health Institute (ISCIII), Madrid, Spain; ^4^ Pneumology Unit, University Clinic Hospital of Valencia, Valencia, Spain; ^5^ CIBERDEM-Spanish Biomedical Research Centre in Diabetes and Associated Metabolic Disorders, Carlos III Health Institute (ISCIII), Madrid, Spain

**Keywords:** cigarette smoking, COPD, GOLD 1, endothelial dysfunction, platelets, leukocytes, systemic inflammation, cytokines

## Abstract

**Background:**

Chronic obstructive pulmonary disease (COPD), usually caused by long-term tobacco smoking, is independently associated with systemic inflammation. However, little is known about the systemic inflammatory status of patients with early-stage COPD (classified as GOLD 1) and long-term smokers with normal lung function (LF). Here, we characterised the early changes in the associated inflammatory state in patients with GOLD 1 and in long-term smokers with normal LF.

**Methods:**

Fresh blood samples from 27 patients with GOLD 1, 27 long-term smokers and 14 non-smokers were analysed.

**Results:**

*Ex vivo* blood analysis revealed greater leucocyte-platelet adhesion to TNFα-stimulated pulmonary endothelium in patients with GOLD 1 than in smokers and non-smokers. In addition, platelet reactivity (platelet count and activation, and fibrinogen levels) and the frequency of leucocyte-platelet aggregates were higher in the GOLD 1 group than in the other groups. Some of these findings correlated with the severity of lung dysfunction, while platelet hyperactivity correlated positively with leucocyte-platelet adhesion. The GOLD 1 group also had a higher Th17/Treg ratio and higher circulating levels of IL-17C and C-reactive protein than the other groups. However, long-term smokers also had higher leucocyte counts and activation, and higher plasma levels of TNFα and IL-6 than non-smokers.

**Conclusion:**

Our data suggest that the altered inflammatory parameters in long-term smokers may represent early biomarkers of COPD. Accordingly, peripheral immune monitoring based on the above parameters may be useful to prevent disease progression in long-term smokers with normal LF and early COPD.

## 1 Introduction

Chronic obstructive pulmonary disease (COPD) is defined as a chronic inflammatory response of the airways and lungs to noxious gases and particles, resulting in progressive and not fully reversible airflow limitation (1). According to the World Health Organisation (WHO), tobacco use is responsible for approximately 70% of COPD cases in developed countries and is the third leading cause of death worldwide (2).

The severity of COPD is defined by the degree of airflow obstruction, according to the GOLD (Global Initiative for Chronic Obstructive Lung Disease) classification, and is categorised as mild (GOLD 1), moderate (GOLD 2), severe (GOLD 3) or very severe (GOLD 4) (3).

Long-term exposure of the lung to external noxious agents induces a chronic proinflammatory state, orchestrated principally by airway epithelial cells and macrophages, which release inflammatory mediators such as interleukin (IL)-1, IL-6, IL-8/CXCL8 and tumour necrosis factor-α (TNFα) into the lung (1). Pulmonary inflammation, in turn, leads to low-grade systemic inflammation involving acute-phase proteins such as C-reactive protein (CRP) and fibrinogen, as well as other cellular and soluble mediators (4, 5). Among the various immune cellular players, neutrophils have been shown to be detrimental in COPD, contributing to tissue destruction through the release of proteases (6). In addition, macrophages exhibit defective phagocytosis and increased production of pro-inflammatory cytokines, playing a complex role in perpetuating chronic inflammation, tissue damage and disease progression (6, 7). Among T lymphocytes, CD8^+^ T cells are the predominant cells in the lungs of patients with COPD and play an important role in the development of emphysema (8). Moreover, the presence of lymphoid follicles, of which B cells are the major component, in the vicinity of the small airways of patients with COPD, suggests that these cells may be relevant to disease development. However, the precise role of immune cells in the development and progression of COPD is not fully understood (6). Beyond traditional immune cells, there is evidence that platelet hyper-reactivity, monocyte-platelet complexes and endothelial dysfunction contribute to the development of COPD and to cardiovascular events in patients with COPD (9), with the caveat that these studies were conducted in patients with more severe airflow obstruction (GOLD 2-4). However, little is known about the systemic inflammatory status in early COPD (10, 11).

Given that low-grade systemic inflammation and pulmonary endothelial dysfunction are key features of COPD (12, 13), the aim of the present study was to comprehensively characterise the peripheral immune system behaviour in patients with mild COPD (GOLD 1) and in long-term smokers without impaired lung function (LF) compared with immune system behaviour in non-smoker controls. Specifically, we investigated leucocyte adhesion to the dysfunctional endothelium to identify potential biomarkers that predict disease exacerbation or the potential development of early COPD.

## 2 Materials and methods

### 2.1 Human study population

Twenty-seven patients with GOLD 1 stage COPD, 27 long-term smokers without COPD (with normal LF) and 14 non-smoker healthy volunteers were included in this cross-sectional study. The recruitment of all participants was made by the Pneumology Unit of the University Clinic Hospital of Valencia. Demographic and clinical features of participants are shown in **Table 1**. Further details, including the *sample size calculation*, can be found in the **Supplementary Material**.

**Table 1:** Demographic and clinical features of participants.

Features	A: Non-smoker volunteers (N = 14)	B: Normal LF smokers (N = 27)	C: GOLD1 patients (N = 27)	P value
**Age (years)**	57.71 ± 1.55	57.59 ± 1.16	60.78 ± 1.35	0.9982 (A *vs.* B) 0.3181 (A *vs.* C) 0.1668 (B *vs.* C)
**Gender M/F (%)**	6/8 (41.9/57.1)	21/6 (77.8/22.2)	14/13 (51.9/48.1)	0.0946 (A *vs.* B) > 0.9999 (A *vs.* C) 0.1601 (B *vs.* C)
**Active Smoking (%)**	N.D.	13 (48.1)	18 (66.6)	0.2709
**CSE (packs-years)**	N.D.	40.96 ± 4.68	39.89 ± 3.44	0.8199
**DLCO (%)**	N.D.	92.74 ± 2.38	70.56 ± 3.70††	**< 0.0001**
**FEV1 (%)**	106.80 ± 3.66	102.40 ± 2.90	93.26 ± 1.68**/†	0.8246 (A *vs.* B) **0.0085 (A *vs.* C)** **0.0382 (B *vs.* C)**
**FEV1/FVC ratio**	77.91 ± 1.47	79.48 ± 0.94	65.07 ± 0.64**/††	0.3087 (A *vs.* B) **< 0.0001 (A *vs.* C)** **< 0.0001 (B *vs.* C)**
**TLC (%)**	N.D.	98.15 ± 2.50	111.30 ± 2.38††	**0.0001**
**IC**	N.D.	110.50 ± 4.25	106.00 ± 3.86	0.4320
**RV (%)**	N.D.	97.96 ± 4.02	138.50 ± 5.99††	**< 0.0001**
**Hb (g/dL)**	14.32 ± 0.44	15.25 ± 0.22	15.03 ± 0.24	0.0999 (A *vs.* B) 0.1966 (A *vs.* C) 0.5399 (B *vs.* C)
**MPV (fL)**	10.79 ± 0.20	10.66 ± 0.20	10.52 ± 0.20	> 0.9999 (A *vs.* B) > 0.9999 (A *vs.* C) 0.9888 (B *vs.* C)
**N/L ratio**	1.51 ± 0.12	1.84 ± 0.14	2.20 ± 0.18*	0.3720 (A *vs.* B) **0.0443 (A *vs.* C)** 0.6542 (B *vs.* C)
**CRP (mg/L)**	1.09 ± 0.18	1.99 ± 0.29	4.84 ± 0.88**/††	0.3710 (A *vs.* B) **0.0011 (A *vs.* C)** **0.0025 (B *vs.* C)**

Data are presented as mean ± SEM. Definition of abbreviations: CRP, c-reactive protein; CSE, cumulative smoking exposure; DLCO, diffusing capacity of the lung for carbon monoxide; FEV1, forced expiratory volume in 1 second; FVC, forced vital capacity; Hb, haemoglobin; IC, inspiratory capacity; LF, lung function; MPV, mean platelet volume; N.D., not determined; N/L, neutrophil-to-lymphocyte; RV, residual volume; TLC, total lung capacity. *P < 0.05 or **P < 0.01 relative to non-smokers’ values. †P < 0.05 or ††P < 0.01 relative to Normal LF smokers’ values.

All p values < 0.05 are highlighted in bold type.

### 2.2 Pulmonary function tests

Pulmonary function tests were performed in the respiratory functional testing laboratory of the University Clinic Hospital (Valencia, Spain). Further details can be found in the **Supplementary Material**.

### 2.3 Biochemical and haematological analyses

Details of the biochemical and haematological analyses can be found in the **Supplementary Material**.

### 2.4 Cell culture

Human pulmonary microvascular endothelial cells (HPMEC) were purchased from Sigma-Aldrich (Madrid, Spain). Details are described in the **Supplementary Material**.

### 2.5 Leucocyte-endothelial cell interactions under flow conditions

To study leucocyte-endothelial cell interaction *ex vivo*, whole blood, previously treated or not with ethylenediaminetetraacetic acid (EDTA), was perfused across endothelial monolayers (HPMEC) unstimulated or stimulated with TNFα (20 ng/mL, Sigma-Aldrich) for 24 hours. Details are described in the **Supplementary Material**.

### 2.6 Flow cytometry

Full details are described in the *Supplemental Material*. Gating strategies are described in **Supplementary e-Figures 1**-**6** and **Supplementary e-Tables 1**, **2** (**Supplementary Material**).

### 2.7 Quantification of soluble inflammatory and metabolic markers

Cytokine profiling was performed using plasma from heparinised whole blood. Further details are found in the **Supplementary Material**.

### 2.8 Statistical analysis

All results were analysed using GraphPad Prism 6 (GraphPad Software, Inc., La Jolla, CA). Values are expressed as individual data points, percentages or mean ± standard error of the mean (SEM) where appropriate. For two-group comparisons, paired or unpaired Student’s *t* test was used for data that passed both normality (*Kolmogorov*-Smirnov*)* and equal variance (Levene) tests, as appropriate; otherwise, the non-parametric Mann-Whitney U-test was performed. For comparisons of multiple groups, one-way analysis of variance followed by *post hoc* Sidak analysis was used for data that passed both normality and equal variance tests; otherwise, the non-parametric Kruskal-Wallis test followed by Dunn’s *post hoc* analysis was used. In all analyses *P* values <0.05 were considered statistically significant. In addition, some correlations between experimental findings and clinical features were calculated using the Pearson and Spearman correlation tests.

## 3 Results

Sixty-eight subjects were recruited in total: 27 patients with GOLD 1, 27 long-term smokers with normal LF and 14 non-smoker controls. Demographic and clinical features of participants are shown in **Table 1**. No significant differences were found in the percentage of active smoking, cumulative smoking exposure or inspiratory capacity between GOLD 1 patients and normal LF smokers, nor in haemoglobin levels or mean platelet volume (MPV) between the three age- and sex-matched groups. Contrastingly, the total lung capacity and residual volume were significantly higher in patients with GOLD 1 than in the other groups, as were CRP levels. Furthermore, the forced expiratory volume in 1 second (FEV1) and the FEV1/forced vital capacity (FVC) ratio were significantly lower in patients with GOLD 1 than in the other groups. Interestingly, CRP levels negatively correlated with FEV1/FVC ratio (**Supplementary e-Figure 7** **).**

### 3.1 Leucocyte-platelet-endothelium interactions are greater in patients with GOLD 1 than in smokers with normal LF and non-smoker controls

**Figure 1:**
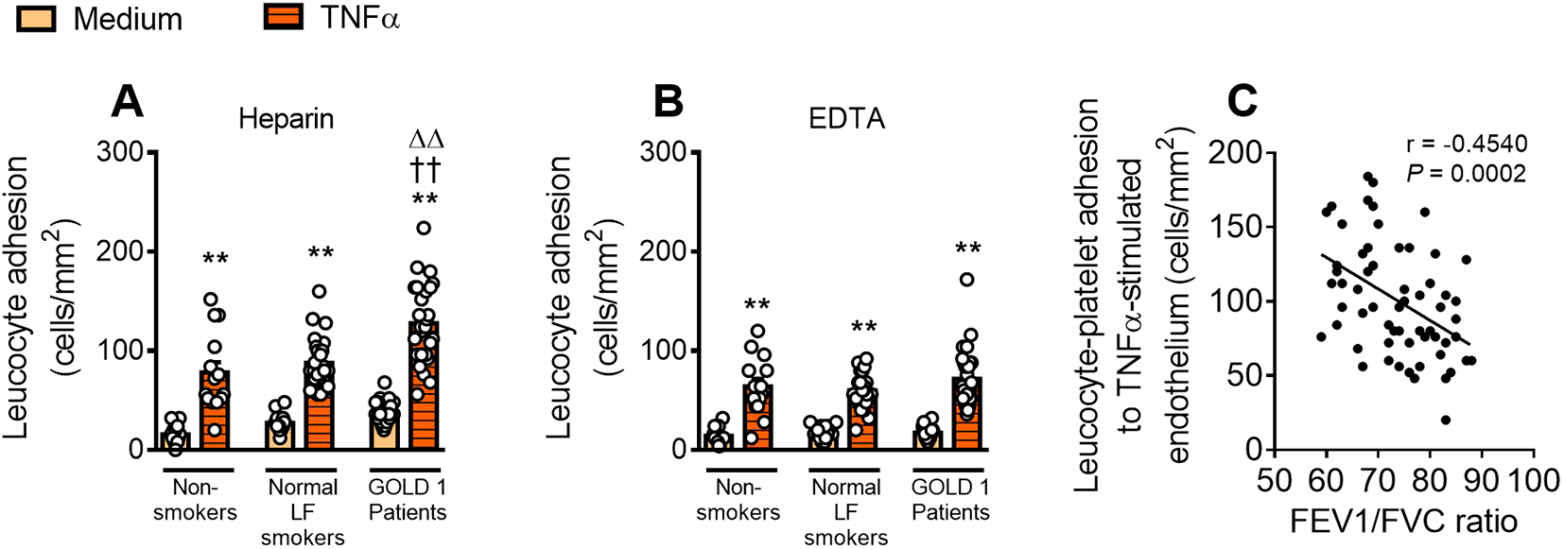
Circulating leucocyte-platelet aggregates from patients with GOLD 1 are more adherent to dysfunctional endothelium. HPMECs were stimulated or not with TNFα (20 ng/mL) for 24 hours. Subsequently, whole blood from subjects, incubated without **(A)** or with **(B)** EDTA, was perfused across endothelial monolayers for 7 minutes at 0.5 dyn/cm^2^ and leucocyte adhesion was quantified. Results are presented as adhered leucocytes per mm^2^ (cells/mm^2^). Values are expressed as mean ± SEM. **P<0.01 relative to values in the respective medium (un-stimulated) group. ††*P*<0.01 relative to respective values in the non-smoker group. ΔΔ*P*<0.01 relative to respective values in the normal LF smokers’ group. Correlations between leucocyte-platelet aggregate adhesiveness to TNFα-induced dysfunctional endothelium and FEV1/FVC ratio **(C)**. FEV1, forced expiratory volume in the first second; FVC, forced vital capacity; HPMECs, human pulmonary microvascular endothelial cells; TNFα, tumour necrosis factor-α.

We first determined the leucocyte-platelet and leucocyte adhesiveness to the dysfunctional pulmonary microvascular endothelium under dynamic flow conditions. Because COPD is associated with low-grade systemic inflammation and elevated circulating levels of TNFα (12, 14, 15), we used this cytokine to mimic endothelial dysfunctionality. HPMEC TNFα stimulation (20 ng/mL, 24 hours) increased leucocyte-platelet and leucocyte endothelial adhesion in all groups (**Figures 1A, B**). Notably, while no differences were observed in platelet-free leucocyte-endothelium interactions between groups (EDTA, **Figure 1B**), circulating leucocyte-platelet aggregates from patients with GOLD 1 showed greater adhesiveness to TNFα-stimulated HPMECs when compared with those of normal LF smokers (45.6% increase) and non-smokers (63.7% increase) (heparin, **Figure 1A**), indicating that platelets likely play a key role in leucocyte-endothelium interactions in early-stage COPD.

Furthermore, leucocyte-platelet aggregate adhesiveness correlated negatively with the FEV1/FVC ratio (**Figure 1C**), a key parameter widely used for COPD diagnosis.

### 3.2 Platelet count and activation, as well as circulating levels of fibrinogen, are significantly higher in patients with GOLD 1 than in normal LF smokers and non-smoker controls

Since platelets appear to play a pivotal role in leucocyte-endothelium interactions in early-stage COPD, we next assessed different platelet-associated parameters.

**Figure 2:**
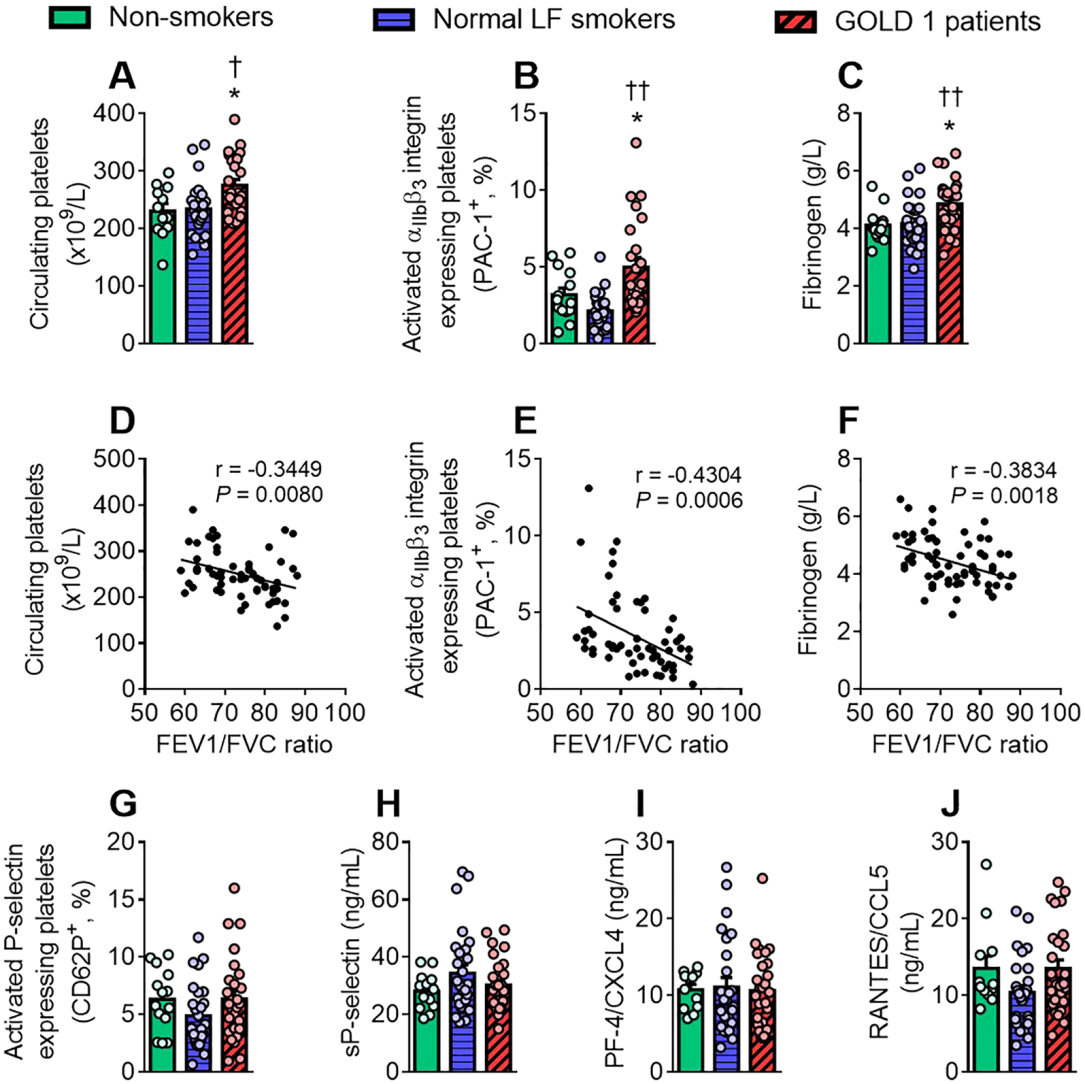
GOLD 1 patients have greater circulating levels of activated platelets and fibrinogen than control groups. Analysis of circulating platelets (×10^9^/L) was determined through a conventional hemogram **(A)**. Flow cytometry analysis of platelets labelled with fluorochrome-conjugated antibodies against CD41 and PAC-1 **(B)**, or CD41 and CD62P/P-selectin **(G)**. Results are presented as the percentage of positive platelets. Soluble (s)P-selectin **(H)**, platelet factor-4 (PF-4)/CXCL4 **(I)** and regulated on activation normal T cell expressed and secreted (RANTES)/CCL5 **(J)** plasma levels (ng/mL) were measured by ELISA, while fibrinogen **(C)** levels were determined by coagulometry (g/L). Values are expressed as mean ± SEM. *P<0.05 relative to values in the respective non-smoker group. †*P*<0.05 or ††*P*<0.01 relative to values in the normal LF smoker group. Correlations between the platelet counts **(D)**, the percentage of PAC-1-expressing platelets **(E)** and the circulating levels of fibrinogen **(F)** and FEV1/FVC ratio. FEV1, forced expiratory volume in the first second; FVC, forced vital capacity.

The absolute count of platelets (**Figure 2A**), the percentage of PAC-1^+^ platelets (**Figure 2B**) and the circulating levels of fibrinogen (**Figure 2C**), were all significantly higher in patients with GOLD 1 than in normal LF smokers and non-smoker controls. All three parameters correlated negatively with the FEV1/FVC ratio (**Figures 2D–F**), suggesting that platelet hyperactivity is intimately related to lung dysfunction in early-stage COPD. Inasmuch, the percentage of PAC-1^+^ platelets and the circulating levels of fibrinogen positively correlated with leucocyte-platelet adhesion to the dysfunctional lung endothelium (**Supplementary e-Figures 8A, B**).

By contrast, no differences between groups were observed in P-selectin platelet expression (CD62P; **Figure 2G**), or in plasma levels of soluble P-selectin (sP-selectin, **Figure 2H**), platelet factor-4 (PF-4)/CXCL4 (**Figure 2I**) and regulated on activation normal T cell expressed and secreted chemokine (RANTES)/CCL5 (**Figure 2J**), or in MPV values (**Table 1**).

### 3.3 Neutrophil counts and granulocyte-platelet aggregate frequencies are significantly higher in patients with GOLD 1 than in normal LF smokers and non-smoker controls

**Figure 3:**
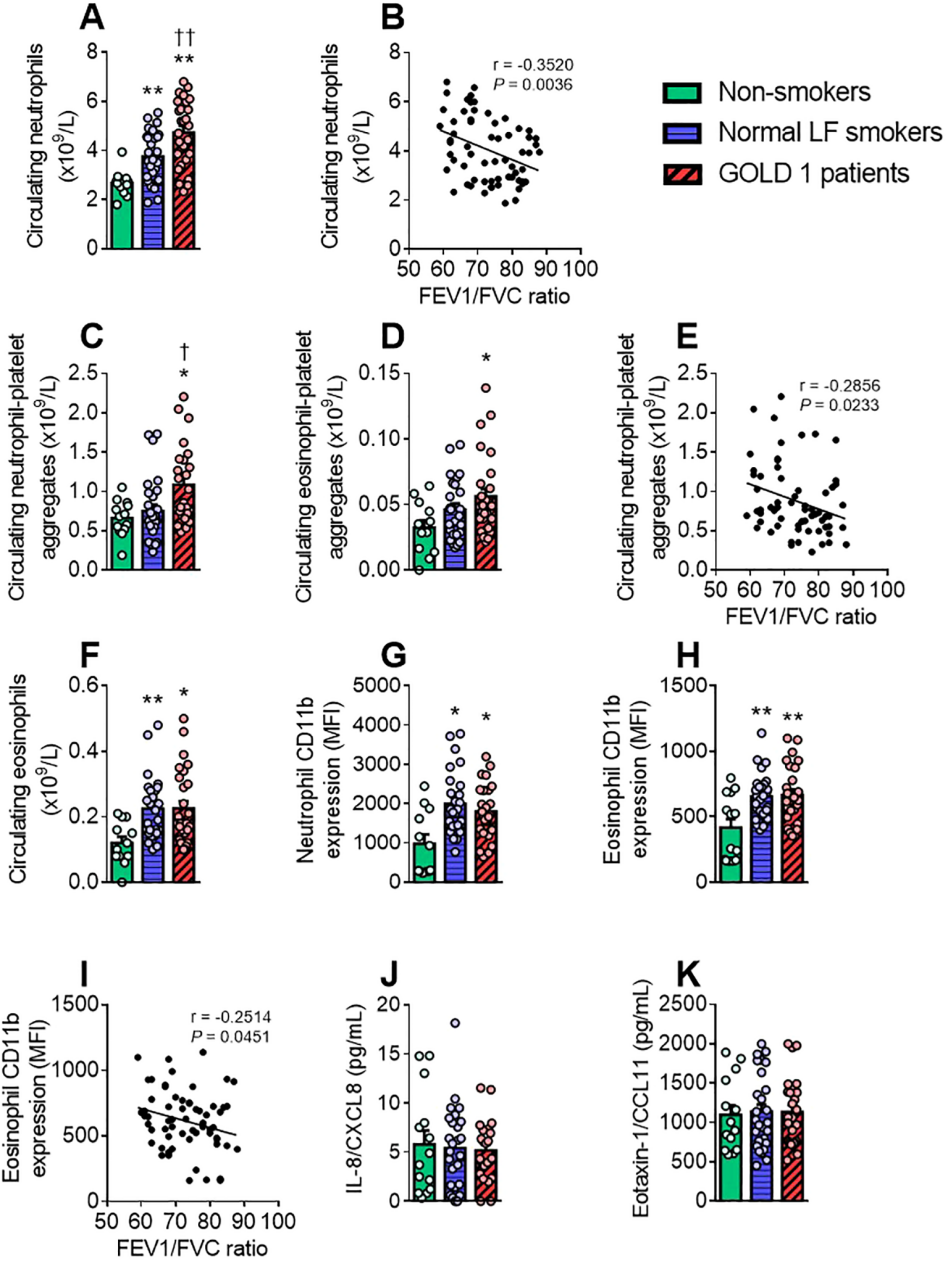
GOLD 1 patients have higher granulocyte counts and activation, and more granulocyte-platelet aggregates than non-smokers. The number of circulating (×10^9^/L) neutrophils **(A)** and eosinophils **(F)** was determined through a conventional hemogram. Flow cytometry analysis of heparinised whole blood co-stained with fluorochrome-conjugated antibodies against specific markers for neutrophils (CD16^+^) or eosinophils (CD16⁻). Neutrophils and eosinophils were also stained with a CD41 **(C, D)** or a CD11b fluorochrome-conjugated antibody **(G, H)**. Results are presented as the number of circulating cells or as the mean fluorescence intensity (MFI). Interleukin (IL)-8/CXCL8 **(J)** and eotaxin-1/CCL11 **(K)** plasma levels (pg/mL) were measured by ELISA. Values are expressed as mean ± SEM. *P<0.05 or **P<0.01 relative to values in the respective non-smoker group. †*P*<0.05 or ††*P*<0.01 relative to values in the normal LF smoker group. Correlations between the neutrophil count **(B)**, the absolute count of neutrophil-platelet aggregates **(E)** or the eosinophil activation **(I)** and FEV1/FVC ratio. FEV1, forced expiratory volume in the first second; FVC, forced vital capacity.

We next investigated several parameters related to the activation state of different leucocyte subpopulations. A hemogram revealed that absolute neutrophil counts were significantly higher in patients with GOLD 1 than in the other groups, and normal LF smokers also showed higher neutrophil counts than non-smokers (**Figure 3A**). Of note, the neutrophil counts correlated negatively with the FEV1/FVC ratio (**Figure 3B**).

Notably, a greater number of circulating neutrophil- and eosinophil-platelet aggregates was found in patients with GOLD 1 than in the other groups (**Figures 3C, D**), and the former was associated with lung dysfunction (**Figure 3E**). Both numbers of leucocyte-platelet aggregates positively correlated with leucocyte-platelet-lung endothelial adhesion (**Supplementary e-Figures 8C, D**).

An analysis of eosinophils revealed higher counts of this leucocyte subset in patients with GOLD 1 and in normal LF smokers than in non-smokers (**Figure 3F**) and, similarly, CD11b expression, an activation marker of neutrophils and eosinophils, was significantly higher in the GOLD 1 and normal LF groups than in the non-smoker group (**Figures 3G, H**). A negative correlation between CD11b expression in eosinophils and the FEV1/FVC ratio was also observed (**Figure 3I**). Finally, no differences were observed in relevant granulocyte-related chemokines such as IL-8/CXCL8 (for neutrophils; **Figure 3J**) and eotaxin-1/CCL11 (for eosinophils; **Figure 3K**) between groups.

### 3.4 Monocyte-platelet aggregate frequency and Mon2 activation are significantly greater in patients with GOLD 1 than in normal LF smokers and non-smoker controls

**Figure 4:**
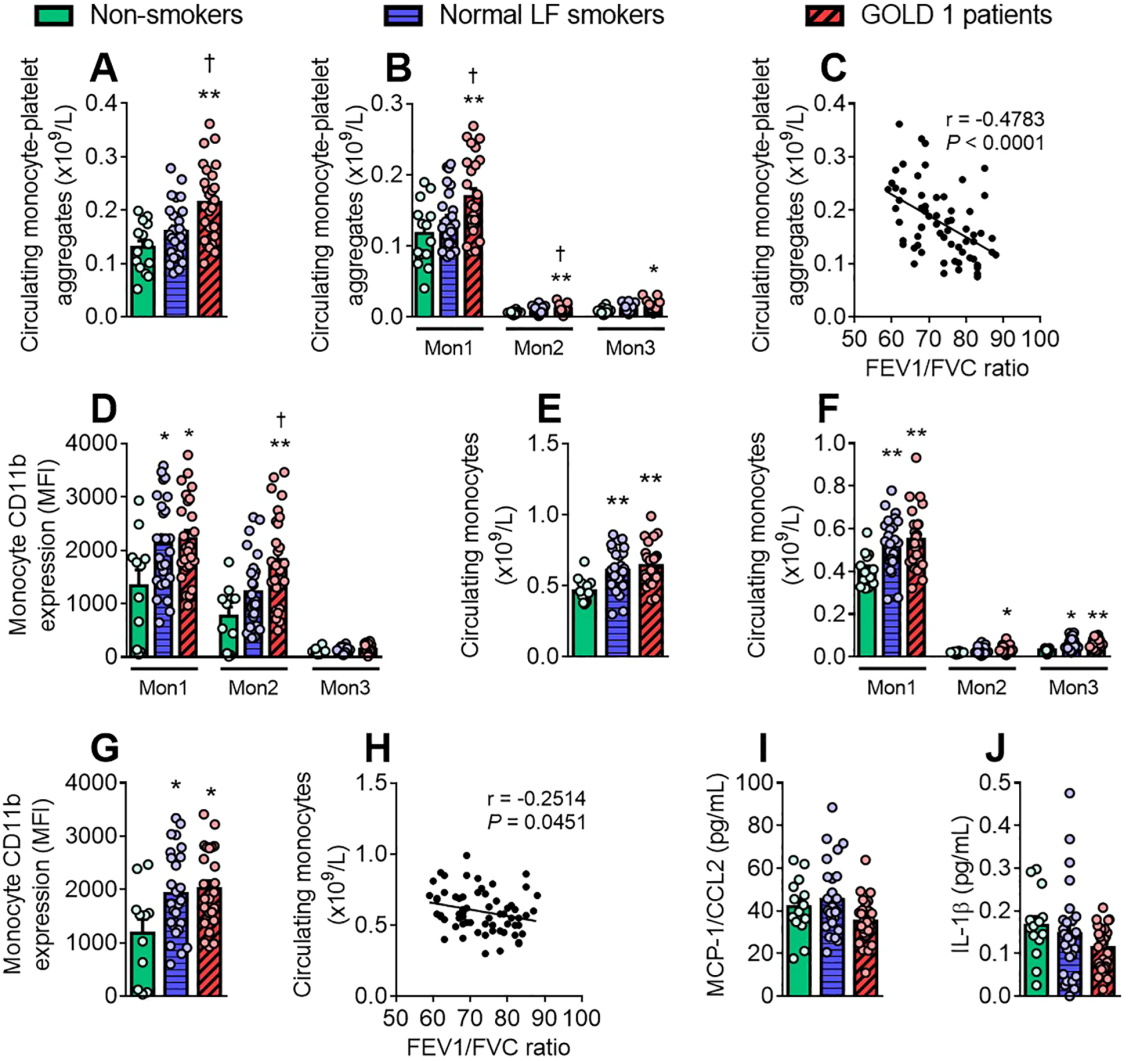
Patients with GOLD 1 show greater monocyte-platelet aggregate formation than normal LF smokers and non-smokers. The number of circulating (×10^9^/L) monocytes **(E, F)** was determined through a conventional hemogram. Flow cytometry analysis of heparinised whole blood co-stained with fluorochrome-conjugated antibodies against specific markers for monocytes (CD14^+^) and the different monocyte subsets (Mon1: CD16^−^CCR2^+^; Mon2: CD16^+^CCR2^+^; Mon3, CD16^+^CCR2^−^). Monocytes were also stained with a CD41 **(A, B)** or a CD11b fluorochrome-conjugated antibody **(D, G)**. Results are presented as the number of circulating cells or as the mean fluorescence intensity (MFI). Monocyte chemoattractant protein-1 (MCP-1)/CCL2 **(I)** and interleukin (IL)-1β **(J)** plasma levels (pg/mL) were measured by ELISA. Values are expressed as mean ± SEM. *P<0.05 or **P<0.01 relative to values in the respective non-smoker group. †*P*<0.05 relative to values in the normal LF smoker group. Correlations between the monocyte count **(H)** or the absolute count of monocyte-platelet aggregates **(C)** and FEV1/FVC ratio. FEV1, forced expiratory volume in the first second; FVC, forced vital capacity.

Monocytes can be divided into three subsets based on the differential expression of surface markers such as CD14, CD16 and CCR2 (**Supplementary e-Table 1**). The number of circulating monocyte-platelet aggregates was significantly higher in the GOLD 1 group than in the other groups (**Figure 4A**), which was due to the contribution of all three monocyte subsets (**Figure 4B**). Again, monocyte-platelet aggregate counts correlated negatively with the FEV1/FVC ratio (**Figure 4C**) and positively with leucocyte-platelet-endothelial adhesion (**Supplementary e-Figure 8E**). CD11b expression was only significantly increased in the intermediate monocyte subpopulation (Mon 2) in the GOLD 1 group (**Figure 4D**).

The absolute count of total monocytes (**Figure 4E**) and monocyte subsets (**Figure 4F**) were greater in both GOLD 1 and normal LF smoker groups than in the non-smoker group. Likewise, monocyte activation (CD11b expression) was higher in both GOLD 1 and normal LF smoker groups than in the non-smoker group (**Figure 4G**). This effect was mainly due to the contribution of the classical monocyte (Mon1) subset (**Figure 4D**). Monocyte counts correlated negatively with the FEV1/FVC ratio (**Figure 4H**). No differences were observed between groups in plasma levels of MCP-1/CCL2 (**Figure 4I**) or the monocyte-derived cytokine IL-1β (**Figure 4J**).

### 3.5 IL-17C circulating levels are significantly greater in patients with GOLD 1 than in normal LF smokers and non-smoker controls

**Figure 5:**
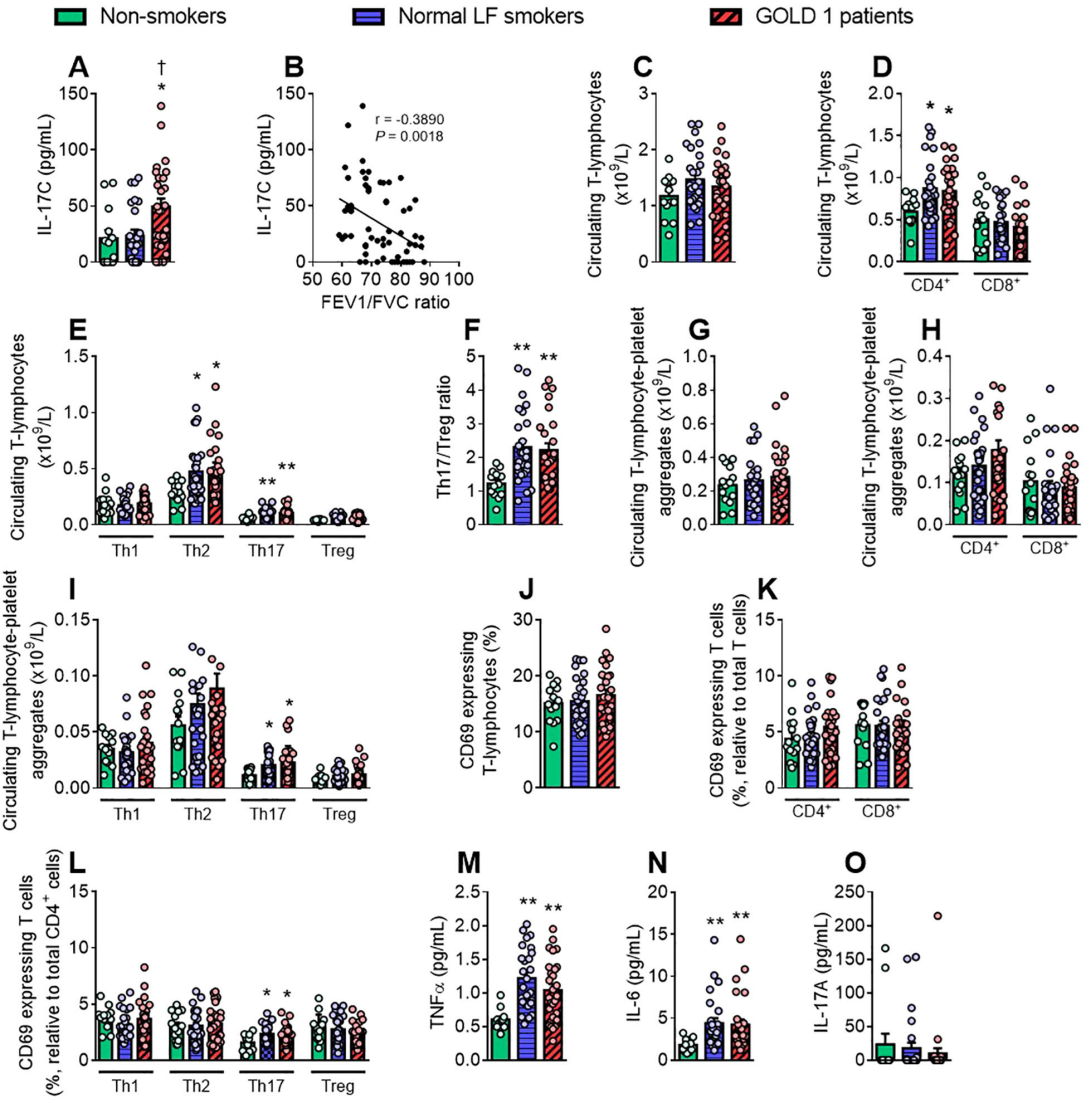
Patients and smokers show increased Th17 cell counts, activation and Th17-platelet aggregates frequency than non-smokers. The number of circulating (×10^9^/L) T-lymphocytes **(C)** and the different T cell subsets **(D, E)** was determined through a conventional hemogram. Th17/Treg ratio was calculated by dividing the absolute count of Th17 cells by the absolute count of regulatory T cells **(F)**. Flow cytometry analysis of heparinised whole blood co-stained with fluorochrome-conjugated antibodies against specific markers for T-lymphocytes (CD3^+^ and CD4^+^ or CD8^+^) and the different CD4^+^ cell subsets (Th1: CXCR3^+^CCR6^−^; Th2: CXCR3^−^CCR6^−^; Th17: CXCR3^−^CCR6^+^; Treg: CD25^+^CD127^low^). T-lymphocytes were also stained with a CD41 **(G–I)** or a CD69 fluorochrome-conjugated antibody **(J, K, L)**. Results are presented as the number of circulating cells or as the percentage of positive cells. Tumour necrosis factor (TNF)-α **(M)**, interleukin (IL)-6 **(N)**, IL-17A **(O)** and IL-17C **(A)** plasma levels (pg/mL) were measured by ELISA. Values are expressed as mean ± SEM. *P<0.05 or **P<0.01 relative to values in the respective non-smoker group. †*P*<0.05 relative to values in the normal LF smoker group. Correlations between IL-17C plasma levels **(B)** and FEV1/FVC ratio. FEV1, forced expiratory volume in the first second; FVC, forced vital capacity.

We next assessed T-lymphocytes (CD3^+^ cells) and their subsets, T helper (Th) cells (CD4^+^ cells) and cytotoxic T cells (CD8^+^ cells) and some related inflammatory soluble markers. Only IL-17C levels were significantly higher in patients with GOLD 1 than in normal LF smokers and non-smoker controls which negatively correlated with the FEV1/FVC ratio (**Figures 5A, B**) but positively with leucocyte-platelet-endothelial adhesion (**Supplementary e-Figure 8G**).

No differences were found in the absolute count of T-lymphocytes and CD8^+^ cells between groups; however, CD4^+^ lymphocyte counts were significantly higher in patients with GOLD 1 and normal LF smokers than in non-smokers (**Figures 5C, D**), which was mainly due to the increased Th2 and Th17 cell counts (**Figure 5E**). Consequently, the Th17/Treg ratio, a marker of systemic inflammation, was also significantly greater in the two former groups than in the non-smoker group (**Figure 5F**).

Both Th17-lymphocyte-platelet aggregate frequencies and Th17 activation (CD69 expression) were greater in patients with GOLD 1 and normal LF smokers than in non-smokers (**Figures 5I, L**). On this matter, no differences were found between groups concerning the other T cell subsets (**Figures 5G, H, J, K**). Accordingly, both GOLD 1 and normal LF smoker groups showed increased plasma levels of TNFα and IL-6 (**Figures 5M, N**). No differences between groups were detected in IL-17A circulating levels (**Figure 5O**).

### 3.6 B-lymphocyte-platelet aggregate frequency is significantly greater in patients with GOLD 1 than in normal LF smokers and non-smokers

**Figure 6:**
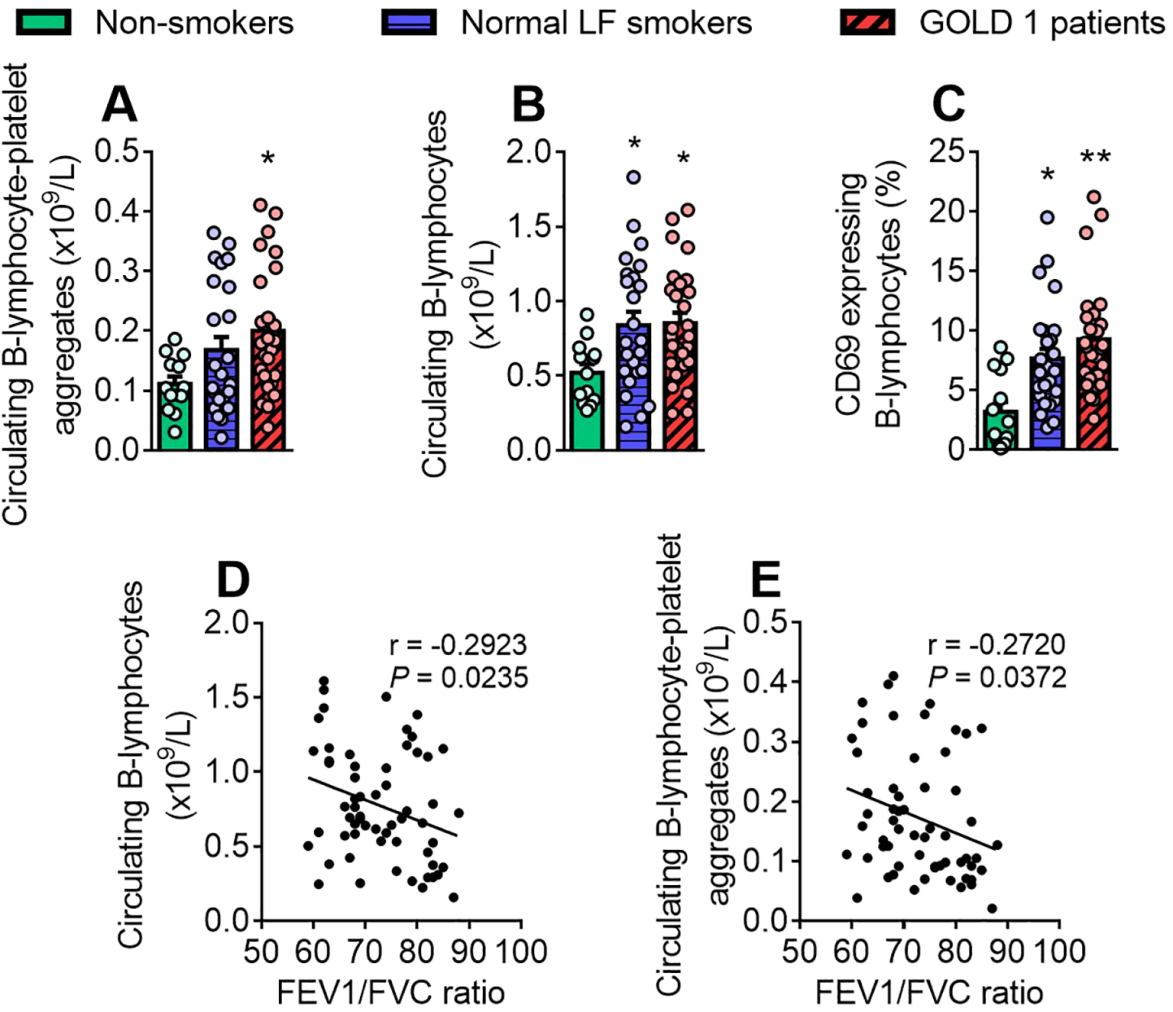
B-lymphocyte-platelet aggregate frequency is significantly greater in patients with GOLD 1 than in non-smokers. The number of circulating B-lymphocytes (×10^9^/L) was determined through a conventional hemogram **(B)**. Flow cytometry analysis of heparinised whole blood co-stained with fluorochrome-conjugated antibodies against specific markers for B-lymphocytes (CD19^+^) and a CD41 **(A)** or a CD69 fluorochrome-conjugated antibody **(C)**. Results are presented as the number of circulating cells or as the percentage of positive cells. Values are expressed as mean ± SEM. *P<0.05 or **P<0.01 relative to values in the respective non-smokers’ group. Correlations between the B-lymphocyte counts **(D)** or the absolute count of B-lymphocyte-platelet aggregates **(E)** and FEV1/FVC ratio. FEV1, forced expiratory volume in the first second; FVC, forced vital capacity.

Finally, we studied the B-lymphocyte population (CD19^+^) in this setting. While B cell-platelet aggregate frequency was greater in the GOLD 1 group than in the non-smoker group (**Figure 6A**), B cell counts and their activation state were higher in both the GOLD 1 and normal LF smoker groups than in the non-smoker group (**Figures 6B, C**). Of note, the absolute count of B-lymphocytes and B-lymphocyte-platelet aggregates (**Figures 6D, E**) correlated negatively with the FEV1/FVC ratio and the latter positively with leucocyte-platelet-endothelial adhesion (**Supplementary e-Figure 8H**).

## 4 Discussion

We demonstrate that the adhesion of circulating leucocyte-platelet aggregates to the dysfunctional pulmonary endothelium is greater in patients with GOLD 1 than in normal LF smokers and non-smoker controls. This was mainly due to a higher number of circulating and activated platelets in the former, as well as to more leucocyte-platelet aggregates, mainly of the myeloid lineage. Of note, these parameters correlated negatively with a key parameter of lung function in COPD, the FEV1/FVC ratio. No correlations were found between these parameters and FEV1, suggesting that the FEV1/FVC ratio is a better indicator of mild COPD (GOLD 1 definition: FEV1/FVC ratio < 0.70 and FEV1 ≥ 80%). Given the finding of no group differences in the adhesion of platelet-free leucocytes to stimulated endothelium, it is likely that platelets play a key functional role in the initial leucocyte arrest in early-stage COPD, a critical event preceding leucocyte infiltration (13). Indeed, enhanced endothelial platelet-unbound leucocyte arrest has only been observed in more severe stages of COPD (GOLD 2-4), likely due to the hyper-inflammatory state of these patients (12, 16).

We found that normal LF smokers and patients with GOLD 1 had elevated levels of circulating neutrophils, eosinophils, monocytes, Th2, Th17 and B cells, which were found in an activated state together with increased plasma levels of TNFα and IL-6 compared with non-smokers. These differences in peripheral immune markers in normal LF smokers may represent a signature of early COPD development before the onset of lung abnormalities.

Consistent with our observations, several studies have highlighted the presence of thrombocytosis and enhanced platelet aggregation in patients with COPD, which has been associated with a higher risk of cardiovascular morbidity and mortality (17, 18). Indeed, we found that absolute platelet counts were higher in patients with GOLD 1 than in normal LF smokers or non-smokers together with evident platelet activation (increased expression of α_IIb_β_3_ integrin, recognised by PAC-1). Of note, α_IIb_β_3_ integrin is the platelet receptor for fibrinogen (19), and elevated circulating fibrinogen levels were also found in patients with GOLD 1. As these parameters correlate negatively with the FEV1/FVC ratio, they may serve as early non-invasive biomarkers of respiratory dysfunction and progression. Contrastingly, no changes were observed between groups in other platelet-related parameters including MPV, which is considered as an indicator of platelet size and function for predicting COPD complications (17), P-selectin/CD62P expression, circulating sP-selectin levels, PF-4/CXCL4 or RANTES/CCL5. This is not surprising given the early stage of the disease, as chemokines such as RANTES/CCL5 have been associated with acute lung inflammation and injury in more severe COPD (20).

We examined the behaviour of different leucocyte subtypes to gain insight into the immune status of early COPD. As a consequence of moderate platelet hyperactivity, patients with GOLD 1 had a higher absolute number of neutrophil-, eosinophil-, monocyte-, Th17- and B cell-platelet aggregates than the other groups. To our knowledge, this is the first report to address this relevant observation, although the presence of a higher percentage of monocyte-platelet aggregates has been described previously, albeit in patients with GOLD 2-4 (10). It is widely accepted that activated platelets can mediate endothelial adhesion of circulating leucocytes, a characteristic feature of dysfunctional endothelium (21, 22). The increased frequency of leucocyte-platelet aggregates described here may explain the increased *in vivo* leucocyte adhesion to dysfunctional pulmonary endothelium found in patients with early COPD, which is likely to be associated with disease progression.

We would like to point out that 30% of the recruited GOLD 1 patients presented eosinophilia, a common subpopulation among some subjects with COPD (23). As expected, significant differences were found in eosinophil-platelet aggregates’ frequencies between eosinophilic COPD and non-eosinophilic COPD patients. However, no significant differences were observed between these two groups regarding the percentage of PAC-1^+^ platelets, the circulating levels of fibrinogen, the circulating neutrophil-, monocyte- and B cell-platelet aggregates frequencies, or leukocyte-platelet aggregates’ adhesiveness to the dysfunctional pulmonary endothelium. We have also analysed possible gender differences. Again, no differences in the above-mentioned parameters were encountered between genders in GOLD 1 patients. Therefore, the platelet hyperactivity found in early-stage COPD seems not to be related to eosinophil counts or patient’s gender.

Analysis of soluble markers of inflammation revealed only two mediators that were significantly higher in patients with GOLD 1: CRP and IL-17C, both of which were negatively correlated with the FEV1/FVC. While elevated CRP is a common marker of systemic inflammation, IL-17C is not. The best characterised IL-17 cytokine is IL-17A, which was originally described as being released by Th17 cells and widely implicated in stable COPD (24–26); however, we found no changes in the plasma levels of this cytokine between the three groups. Contrastingly, we found that circulating IL-17C levels were significantly elevated only in those patients with early COPD. IL-17C is predominantly expressed by epithelial cells on the mucosal surfaces of the respiratory tract. It can also stimulate the release of cytokines such as IL-1β, TNF-α and IL-6, which enhance inflammatory responses in the lung (27). In addition, IL-17C is partly responsible for the recruitment and accumulation of neutrophils in the lung, a hallmark of COPD, and may be involved in the pathogenesis of COPD exacerbations through the synthesis of other cytokines by interacting with IL-17RE present on Th17 lymphocytes (28). This may explain the increased neutrophil counts and Th17 activation seen in the GOLD 1 group, leading to an increased neutrophil-to-lymphocyte ratio.

It is pertinent to mention the correlations found between the results and the adhesiveness of circulating leucocyte-platelet aggregates to the dysfunctional endothelium. First, the results suggest a close relationship between platelet hyperreactivity (as evidenced by high platelet expression of α_IIb_β_3_ integrin, circulating levels of fibrinogen and leucocyte-platelet aggregation) and increased endothelial adhesiveness of leucocyte-platelet aggregates. Second, IL-17C positively correlated with leucocyte-platelet adhesion to the dysfunctional lung endothelium, reinforcing the idea that peripheral inflammatory markers such as those described here may be an attractive target to prevent early COPD to more severe states.

Several inflammatory parameters differed between the non-smoker group and both normal LF smokers and patients with GOLD 1, with no significant differences between the latter two groups. In particular, the absolute numbers of neutrophils, eosinophils, monocytes (including all monocyte subsets), Th2, Th17 and B cells were significantly higher in normal LF smokers and patients with GOLD 1 than in non-smokers. Increased leucocyte counts have been widely described in COPD, especially in more severe stages, leading to increased leucocyte infiltration in the lungs of these patients (6, 10, 29). However, long-term smokers also showed abnormalities in hemogram parameters such as increased neutrophil, eosinophil, monocyte and lymphocyte counts, which are potential indicators of systemic inflammation and can be used to predict morbidity and mortality in other diseases (30, 31). Of note, in our comprehensive immunophenotype analysis, we found that the increased number of circulating lymphocytes in normal LF smokers and patients with GOLD 1 was mainly due to the increased levels of Th2, Th17 and B cells, but not those more closely associated with moderate/severe COPD and its exacerbations such as CD8^+^, Th1 or Treg cells (6), which has not been previously addressed.

Consistent with these observations, most of the aforementioned leucocyte subpopulations were in a more activated state in normal LF smokers and patients with GOLD 1 than in non-smokers (e.g., increased CD11b expression in the myeloid lineage and CD69 expression in Th17 and B cells). Interestingly, some of these parameters correlated negatively with the FEV1/FVC ratio. Taken together, it seems that both the increased number of the different leucocyte subpopulations and their activation state may favour the infiltration of these subsets into the lung and contribute to the initial dysfunction of the pulmonary environment.

Among the soluble markers examined, elevated circulating levels of both TNFα and IL-6 were found in normal LF smokers and patients with GOLD 1. TNFα and IL-6 are well-known cytokines produced by a variety of immune and non-immune cells, including Th17 lymphocytes (32), whose number and activation state are increased in both normal LF smokers and patients with GOLD 1. Additionally, the Th17/Treg ratio was elevated in these two groups. Th17 cells suppress Treg function and contribute to a pro-inflammatory milieu. It is therefore tempting to speculate that there is a conversion of Treg cells into Th17 cells in a smoking or early COPD environment. Accordingly, the above parameters could be potential biomarkers to predict the high risk of COPD in asymptomatic smokers.

In conclusion, we have characterised the early changes in the COPD-associated inflammatory state as well as those in normal LF smokers. Although GOLD 1 patients and long-term smokers with normal functions generally presented similar values of leukocyte counts, leukocyte activation, and TNFα or IL-6 plasma levels, our results indicate a remarkable platelet hyperactivity in early-stage COPD, which appears to be responsible for the increased frequency of leucocyte-platelet aggregates and their subsequent adhesion to the dysfunctional pulmonary endothelium. In this sense, we speculate that antiplatelet therapy with low-dose of aspirin may prevent the initial infiltration of leucocytes into the lung, thereby limiting disease progression. Additionally, platelet activation (PAC-1^+^), circulating levels of fibrinogen and IL-17C and the absolute number of leucocyte-platelet aggregates may be biomarkers for predicting disease progression in early COPD. Finally, soluble markers such as circulating TNFα and IL-6, as well as leucocyte count and activation state, emerge as potential biomarkers of early COPD development in long-term smokers with normal LF.

## Data Availability

The raw data supporting the conclusions of this article will be made available by the authors, without undue reservation.
