# Supplementary material for: Key role of activated platelets in the enhanced adhesion of circulating leucocyte-platelet aggregates to the dysfunctional endothelium in early-stage COPD
Source: Front Immunol. 2024 Aug 19;15:1441637. doi: 10.3389/fimmu.2024.1441637 (PMC11369892; doi:10.3389/fimmu.2024.1441637)
Supplement: Supplementary file 1 [file DataSheet1.pdf]

Supplementary Material

1 Sample size calculation

The study by Vestbo *et al.* followed patients from the ECLIPSE study for 3 years and observed that 38% of COPD patients showed disease progression, measured by FEV1 declined[1]. In a previous study conducted in New York (USA) with smokers showing no alteration in lung function tests, it was observed that 2% of patients developed COPD after a 3-year follow-up period[2]. Therefore, the sample size calculation is justified as follows: accepting an alpha risk of 0.05 and a beta risk of 0.2 in a bilateral test, 27 subjects are required in both the first and second groups to detect that the difference between two proportions is statistically significant, expected to be 0.02 for group 1 and 0.38 for group 2. A follow-up loss rate of 10% has been estimated. The ARCSINE approximation has been used.

2 Human study population

To characterise the early changes in the COPD-associated inflammatory state, 27 GOLD 1 patients, 27 long-term smokers without COPD (with a normal lung function) and 14 non-smoker healthy volunteers were recruited, and fresh heparinized- and citrated-blood samples were collected and analysed. The recruitment of all participants was made by the Pneumology Unit of the University Clinic Hospital of Valencia (Valencia, Spain) and was opportunistic.

The eligibility criteria for the present study were as follows. The patients must meet all the inclusion criteria and none of the exclusion criteria.

The inclusion criteria were:

- Mild-COPD diagnosis (GOLD 1): First, chronic obstructive pulmonary disease was diagnosed based on clinical criteria with confirmation of irreversible obstruction in a functional test (spirometry) based on the Global Initiative for Chronic Obstructive Lung Disease (GOLD) 2023 guidelines[3] by a baseline post-bronchodilator Forced Expiratory Volume in 1 second (FEV1)/Forced Vital Capacity (FVC) < 0.7. Then, Mild-COPD was diagnosed when FEV1 > 80%.
- Long-term smokers with normal lung function: Current or former smoking history of ≥ 10 pack-years with FEV1/FVC > 0.7 and diffusing capacity of the lung for carbon monoxide (DLCO) > 80%.
- Non-smoker healthy volunteers with normal lung function: Non-smokers with FEV1/FVC > 0.7.

The exclusion criteria were:

1. Concomitant diagnosis of asthma;
2. History of inflammatory disease (rheumatoid arthritis, Crohn’s disease, etc.);
3. Use of anti-inflammatory drugs in the last 6 weeks.

The study complied with the principles outlined in the Declaration of Helsinki and was approved by the institutional ethics committee of the University Clinic Hospital of Valencia (Valencia,

Spain). All patients signed an informed consent. Demographic and clinical features of participants are shown in **Table 1** of the manuscript.

3 Pulmonary function tests

Pulmonary function tests (PFT) were performed in the respiratory functional testing laboratory of the University Clinic Hospital (Valencia, Spain), and included the determination of lung volumes [total lung capacity (TLC), residual volume (RV), inspiratory capacity (IC) using plethysmography plethysmography (Masterscreen PFT Body, Jaeger, Wuppertal, Germany)], and spirometry [forced vital capacity (FVC), forced expiratory volume in 1 second (FEV1) and FEV1/FVC ratio, using a spirometer (Masterscreen PFT Body, Jaeger)]. The diffusing capacity of the lung for carbon monoxide (DLCO) was determined by the single breath technique using an infrared analyser (Masterscreen PFT Body, Jaeger), and then adjusted for haemoglobin values. All procedures were performed according to American Thoracic Society (ATS) and European Respiratory Society (ERS) guidelines[4].

4 Biochemical and haematological analyses

The number of circulating ($\times 10^9$/L) platelets and leucocyte subsets (neutrophils, eosinophils, monocytes and lymphocytes) was determined through a conventional haemogram using laser flow cytometry (Sysmex XN-9000, Sysmex, Kobe, Japan). Additionally, through the percentages obtained from the flow cytometry methodology described in the section ***Flow cytometry***, the absolute count of different monocyte and lymphocyte subsets, as well as the absolute count of leucocyte-platelet aggregates were calculated.

Furthermore, the neutrophil-to-lymphocyte ratio, one of the proposed prognostic markers of COPD progression[5], was calculated by dividing the absolute count of neutrophils by the absolute count of lymphocytes. Similarly, given that Th17/Treg imbalance in COPD has been described[6], Th17/Treg ratio was calculated by dividing the absolute count of T helper 17 cells by the absolute count of regulatory T cells.

Finally, several biochemical parameters, including fibrinogen (g/L), CRP (mg/L), haemoglobin (g/dL) and mean platelet volume (MPV, fL), were measured by coagulometry or immunoturbidimetry (Beckman Coulter Au5800, Beckman Coulter, Pasadena, CA).

5 Cell culture

Human pulmonary microvascular endothelial cells (HPMEC) were purchased from Sigma-Aldrich (Madrid, Spain) and were maintained in human endothelial cell specific medium (EBM-2, Lonza, Barcelona, Spain) supplemented with endothelial growth media (EGM-2, Lonza) and 10% fetal bovine serum (FBS, Biowest, Nuaillé, France). Prior to every experiment, cells were incubated for 24 h in medium containing 2% FBS.

6 Leucocyte-endothelial cell interactions under flow conditions

Before starting each assay, Hank's balanced salt solution (HBSS, Lonza, Barcelona, Spain) was perfused at 37ºC in order to adjust the flow rate to 0.156 mL/min (corresponding to a control shear stress of 0.5 dyn/cm^2). Then, the circular flow chamber (for 35mm tissue culture dishes; GlycoTech, Rockville, MD) was assembled and placed onto an inverted microscope stage.

To study leucocyte-endothelium interactions *ex vivo*, whole blood from patients (diluted 1:10 in HBSS) was perfused, using a Harvard PHD 22/2000 Advance Syringe Pump (Harvard Apparatus, Cambridge, MA), across 35mm tissue culture dishes-containing HPMEC monolayers previously stimulated or not with 20 ng/mL TNFα (Sigma-Aldrich, Madrid, Spain) for 24 h. Again, experiments were performed using heparinized blood treated or not with EDTA (10 mM, for 15 min, 37ºC) to evaluate the contribution of platelets to leucocyte adhesion[7, 8].

Leucocyte interactions were determined after 7 min at 0.5 dyn/cm^2. Cells interacting on the surface of the endothelium were visualized and recorded (×20 objective, ×10 eyepiece) using phase-contrast microscopy (Axio Observer A1 Carl Zeiss microscope; Carl Zeiss, Thornwood, NY). For each determination, at least 5 fields were recorded for 10 s and then averaged. Finally, recorded images were saved on a computer for further analysis.

7 Flow cytometry

Fresh blood samples from patients were collected in BD Vacutainer blood collection tubes (BD Biosciences, San Jose, CA) containing sodium citrate (3.2%), or in BD Vacutainer PST II tubes with lithium/heparin as anticoagulant agents (17 IU/mL; BD Biosciences).

A total of 50 µL of blood was used for leucocyte immunophenotyping, and 20 µL was used for platelet immunophenotyping. Saturated amounts of fluorochrome-conjugated monoclonal antibodies (mAb) were added to blood samples, along with 50 µL of brilliant stain buffer (BD Biosciences). Each sample was gently shaken and incubated for 30 min at room temperature in the dark. Then, 1× lysis buffer (BD Phosflow™ Lyse/Fix Buffer 5× concentrate, BD Biosciences) was added to each tube to lyse erythrocytes. Subsequently, all samples were run in a BD LSRFortessa™ X-20 flow cytometer (BD Biosciences).

To determine platelet activation, the percentages of PAC-1$^+$ (detects activated integrin $\alpha_{IIb}\beta_3$) and P-selectin$^+$ (CD62P) platelets were measured in subjects' samples by flow cytometry. Citrated blood samples (6.25 µL), diluted 1:10 in glucose buffer (1 mg/mL glucose in PBS containing 0.35% bovine serum albumin [BSA], Sigma-Aldrich), were incubated in the dark for 30 minutes with a 5-carboxyfluorescein (CF)-Blue™-conjugated mAb against human CD41 (1.25 µL, clone HIP8, IgG1, Immunostep, Salamanca, Spain) and a fluorescein isothiocyanate (FITC)-conjugated mAb against the human integrin $\alpha_{IIb}\beta_3$/GPIIb/IIIa (2.5 µL, clone PAC-1, IgM; BD Biosciences) or with an allophycocyanin (APC)-conjugated mAb against human P-selectin (1.25 µL, clone HI62P, IgG1, Immunostep). The CD41$^+$ population (platelets) was selected according to the gating strategy illustrated in **e-Figure 1** and expressed as the percentage of positive platelets.

To identify the different leucocyte subsets, fluorochrome-labelled antibodies against different surface markers were employed. Blood samples were incubated with an FITC-conjugated mAb against human CD16 (5 µL, clone 3G8, IgG1, BD Biosciences) to detect neutrophils (CD16$^+$, **e-Figure 2**) and eosinophils (CD16$^-$, **e-Figure 2**). For total monocytes (CD14$^+$, **e-Figure 3**), a brilliant violet™ (BV)650-conjugated mAb against human CD14 (1.25 µL, clone M5E2, IgG2A, BD Biosciences) was

employed. An FITC-conjugated mAb against human CD16 (5 μL, clone 3G8, IgG1, BD Biosciences) and a BV510-conjugated mAb against human CCR2 (2.5 μL, clone K036C2, IgG2A, BioLegend, San Diego, CA) were used to detect classical monocytes (Mon1, $CD14^{++}CD16^{-}CCR2^{+}$, **e-Table 1, e-Figure 3**), intermediate monocytes (Mon2, $CD14^{++}CD16^{+}CCR2^{+}$, **e-Table 1, e-Figure 3**) or nonclassical monocytes (Mon3, $CD14^{+}CD16^{+}CCR2^{-}$, **e-Table 1, e-Figure 3**). For T-lymphocytes, an APC-H7-conjugated mAb against human CD3 (1.25 μL, clone SK7, IgG1, BD Biosciences, **e-Figure 4**) was employed. Additionally, a brilliant ultraviolet™ (BUV)395-conjugated mAb against human CD4 (1.25 μL, clone RPA-T4, IgG1, BD Biosciences) was used for T-helper cells ($CD3^{+}CD4^{+}$, **e-Figure 4**) detection and an FITC-conjugated mAb against human CD8 (10 μL, clone RPA-T8, IgG1, BD Biosciences) was used for cytotoxic T-cells ($CD3^{+}CD8^{+}$, **e-Figure 4**). For the different Th cell subsets, a brilliant ultraviolet™ (BUV)395-conjugated mAb against human CD4 (1.25 μL, clone RPA-T4, IgG1, BD Biosciences), an APC-conjugated mAb against human CXCR3 (10 μL, clone 1C6/CXCR3, IgG1, BD Biosciences) and a phycoerythrin (PE)/Cy7-conjugated mAb against human CCR6 (2.5 μL, clone 11A9, IgG1, BD Biosciences) were employed as follows: T-helper 1 cells (Th1, $CD4^{+}CXCR3^{+}CCR6^{-}$, **e-Table 2, e-Figure 4**), T-helper 2 cells (Th2, $CD4^{+}CXCR3^{-}CCR6^{-}$, **e-Table 2, e-Figure 4**) and T-helper 17 cells (Th17, $CD4^{+}CXCR3^{-}CCR6^{+}$, **e-Table 2, e-Figure 4**). Furthermore, to detect regulatory T-cells (Treg, $CD4^{+}CD25^{+}CD127^{low}$, **e-Figure 5**), we used a BUV395-conjugated mAb against human CD4 (1.25 μL, clone RPA-T4, IgG1, BD Biosciences), an APC-conjugated mAb against human CD25 (10 μL, clone M-A251, IgG1, BD Biosciences) and a BV650-conjugated mAb against human CD127 (2.5 μL, clone HIL-7R-M21, IgG1, BD Biosciences). Finally, to assess B-lymphocyte population ($CD19^{+}$ population, **e-Figure 6**), a FITC-conjugated mAb against human CD19 (2.5 μL, clone SJ25C1, IgG1, BioLegend) was employed. Gating strategies are illustrated in **e-Figures 2–6**.

To determine leucocyte subset activation, the expression of CD11b was analysed on circulating neutrophils, eosinophils and monocyte subsets, or CD69 expression on T-lymphocyte subsets and B-lymphocytes. Heparinized whole blood samples were incubated in the dark for 30 min with saturated amounts of an APC-conjugated mAb against human integrin CD11b (2.5 μL, clone M1/70, IgG2B; BD Biosciences) or a BV711-conjugated mAb against human CD69 (2.5 μL, clone FN50, IgG1; BioLegend). In some experiments, heparinized blood samples were incubated with EDTA (10 mM, for 15 min at 37ºC) to promote platelet dissociation, as described[7, 8]. This disaggregation was measured using the CD41 marker in circulating leucocyte subsets. To do this, heparinized whole blood or EDTA samples were incubated in the dark for 30 min with saturated amounts of a PerCP-Cy™5.5-conjugated mAb against human CD41 (2.5 μL, clone HIP8, IgG_1; BD Biosciences). Red blood cells were lysed using a commercial lysis buffer (BD Phosflow™ Lyse/Fix Buffer 5× concentrate, BD Biosciences).

All blood samples were run in a BD LSRFortessa™ X-20 flow cytometer (BD Biosciences). All flow cytometry data were analysed using FlowJo® v10.0.7 software (FlowJo LLC, Ashland, OR).

8 Quantification of soluble inflammatory and metabolic markers

The following soluble markers were measured in plasma samples by enzyme-linked immunosorbent assay (ELISA; DuoSet® or Quantikine® ELISA Kits, R&D Systems, Abingdon, UK) as previously described[8]: human soluble IL-1β, IL-6, IL-10, IL-17A, IL-17C, TNFα, platelet factor-4 (PF-4/CXCL4), IL-8/CXCL8, monocyte chemoattractant protein-1 (MCP-1/CCL2), regulated on activation normal T cell expressed and secreted chemokine (RANTES/CCL5), eotaxin-1/CCL11 and soluble P-selectin (sP-selectin). Results were expressed as pg or ng/mL of mediator in plasma.

9 Supplementary Figures and Tables

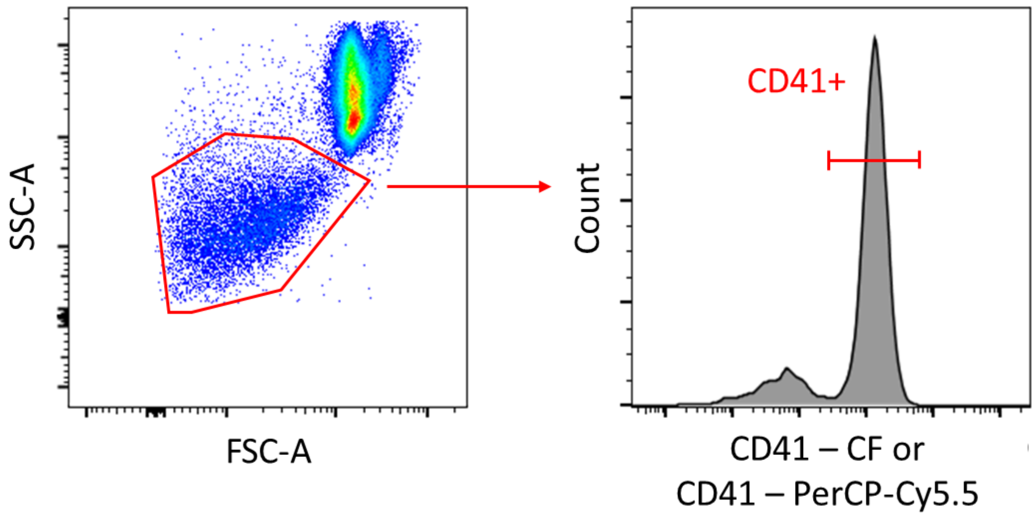

e-Figure 1. Gating strategy for human platelets in whole blood according to morphological properties and CD41 detection by flow cytometry. Platelets were gated according to a low side scatter (SSC-A) and forward scatter (FSC-A) in a logarithmic scale and defined as $CD41^{+}$ population.

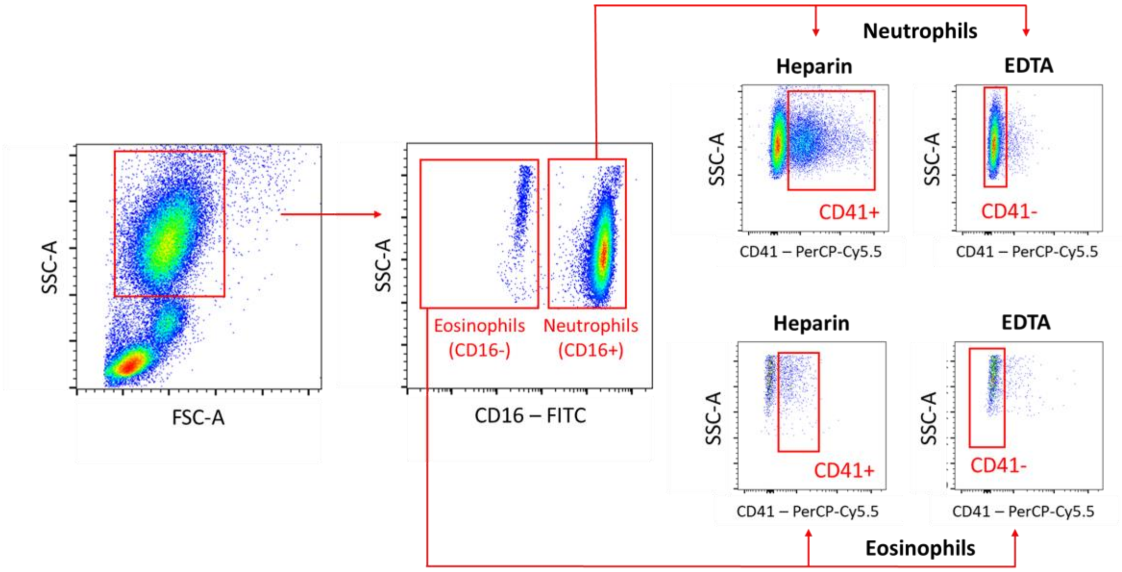

e-Figure 2. Gating strategy for human neutrophils and eosinophils in whole blood according to morphological properties and CD16 expression by flow cytometry. Populations were selected by morphology (high SSC-A). A CD16 antibody was used to detect neutrophils ($CD16^+$) and eosinophils ($CD16^–$). In heparinized blood, neutrophil-platelet-aggregates were selected as a $CD16^+CD41^+$ population and eosinophil-platelet aggregates as a $CD16^–CD41^+$ population; whereas platelet-free neutrophils were gated as a $CD16^+CD41^–$ population and platelet-free eosinophils as a $CD16^–CD41^–$ population from blood incubated with EDTA.

e-Table 1. Differential markers of monocyte subpopulations

Marker	Cellular population
**$CD14^{++}CD16^{-}CCR2^{+}$**	Monocyte type 1 (Mon1)
**$CD14^{++}CD16^{+}CCR2^{+}$**	Monocyte type 2 (Mon2)
**$CD14^{+}CD16^{+}CCR2^{-}$**	Monocyte type 3 (Mon3)

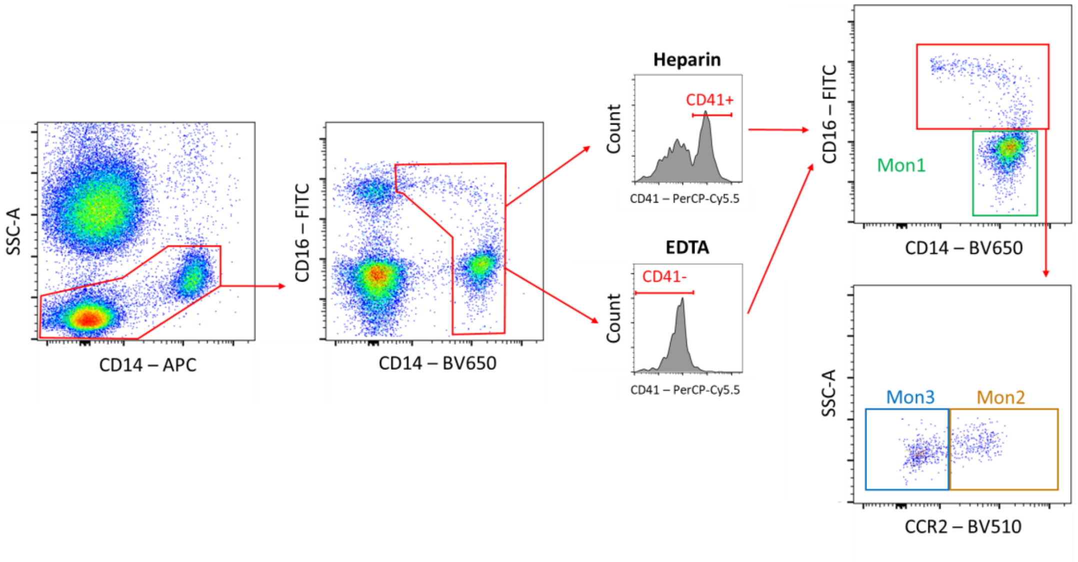

e-Figure 3. Gating strategy for human monocyte detection in whole blood by flow cytometry. Monocytes were selected by CD14 labelling and morphology (medium SSC-A). For the detection of monocyte subpopulations, CD16 and CCR2 markers were used. Monocyte-platelet complexes were selected as $CD14^{+}CD41^{+}$ populations in heparinized whole blood, and platelet-free monocytes were gated as $CD14^{+}CD41^{-}$ populations from blood incubated with EDTA.

e-Table 2. Differential markers of T-helper subpopulations

Marker	Cellular population
**$CD4^+CXCR3^+CCR6^-$**	T helper 1 (Th1)
**$CD4^+CXCR3^-CCR6^-$**	T helper 2 (Th2)
**$CD4^+CXCR3^-CCR6^+$**	T helper 17 (Th17)

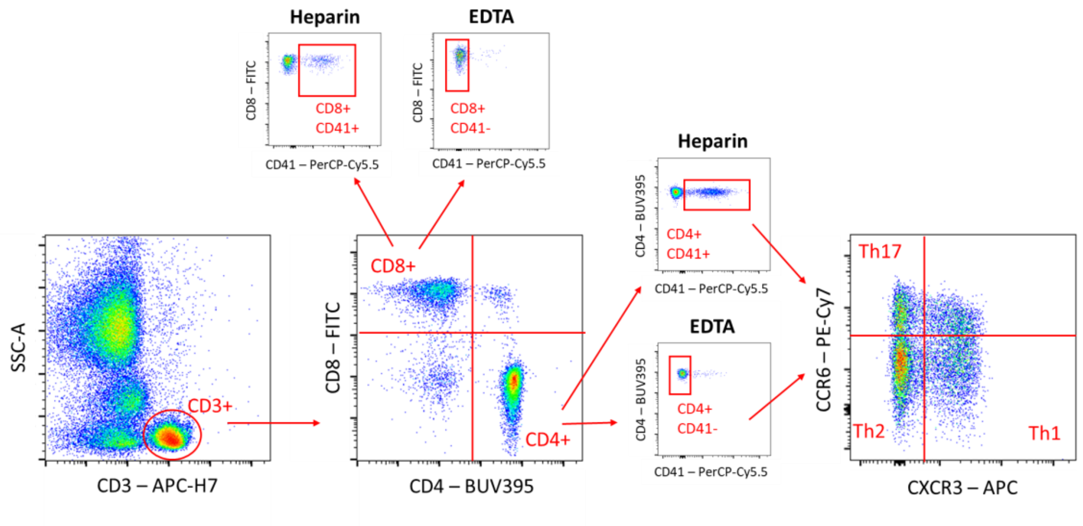

e-Figure 4. Gating strategy for human T-lymphocyte detection in whole blood by flow cytometry. T-lymphocytes were selected as a $CD3^+$ population and with a low SSC-A. Cytotoxic lymphocytes were selected as $CD3^+CD8^+$. In heparinized blood, cytotoxic lymphocyte-platelet complexes were selected as the $CD3^+CD8^+CD41^+$ population, whereas platelet-free cytotoxic lymphocytes were gated as $CD3^+CD8^+CD41^-$ from blood incubated with EDTA. T-helper (Th) lymphocytes were selected as the $CD3^+CD4^+$ population. In heparinized blood, Th lymphocyte-platelet complexes were selected as the $CD3^+CD4^+CD41^+$ population, whereas platelet-free Th lymphocytes were gated as a $CD3^+CD4^+CD41^-$ population from blood incubated with EDTA. Th lymphocyte subpopulations were detected with the markers CXCR3 and CCR6. In heparinized blood, Th1 lymphocyte-platelet complexes were selected as $CD4^+CXCR3^+CCR6^-CD41^+$, Th2 lymphocyte-platelet complexes were selected as $CD4^+CXCR3^-CCR6^-CD41^+$ and Th17 lymphocyte-platelet complexes were selected as $CD4^+CXCR3^-CCR6^+CD41^+$; whereas platelet-free Th lymphocyte subpopulations were gated as a $CD4^+CXCR3^+CCR6^-CD41^-$ (Th1), a $CD4^+CXCR3^-CCR6^-CD41^-$ (Th2) or a $CD4^+CXCR3^-CCR6^+CD41^-$ (Th17) population from blood incubated with EDTA.

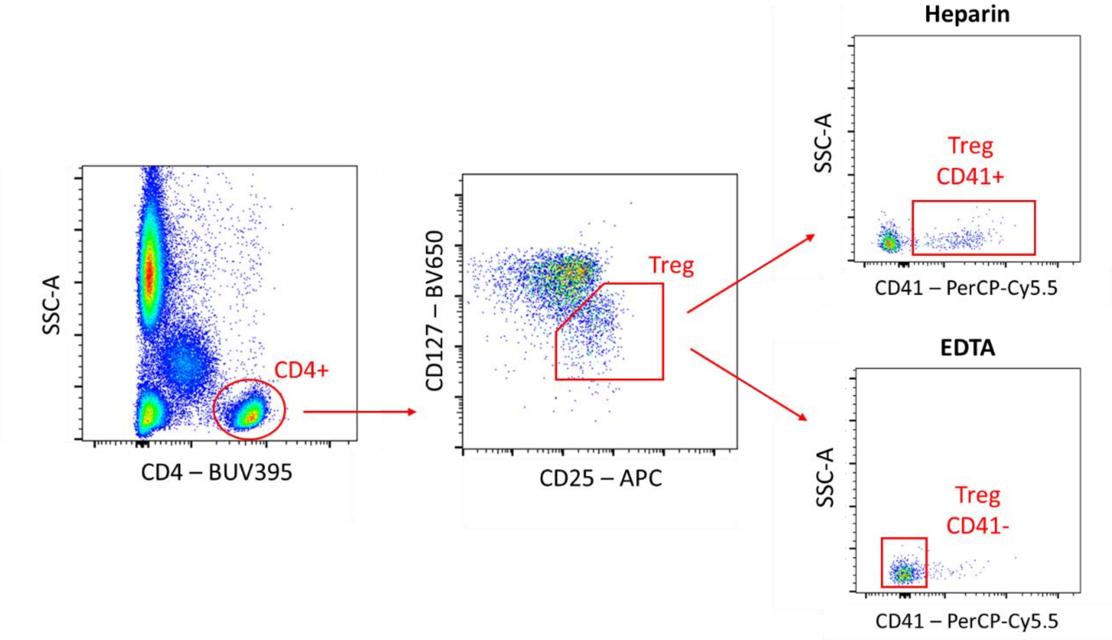

e-Figure 5. Gating strategy for human regulatory T-lymphocyte (Treg) detection in whole blood by flow cytometry. Treg lymphocytes were selected as the CD4+ population and with a low SSC-A. Treg lymphocytes were detected with the markers CD127 and CD25. Treg lymphocyte-platelet complexes were selected as the $CD4^{+}CD127^{low}CD25^{+}CD41^{+}$ population from heparinized whole blood, whereas platelet-free Treg lymphocytes were gated as a $CD4^{+}CD127^{low}CD25^{+}CD41^{-}$ population from blood incubated with EDTA.

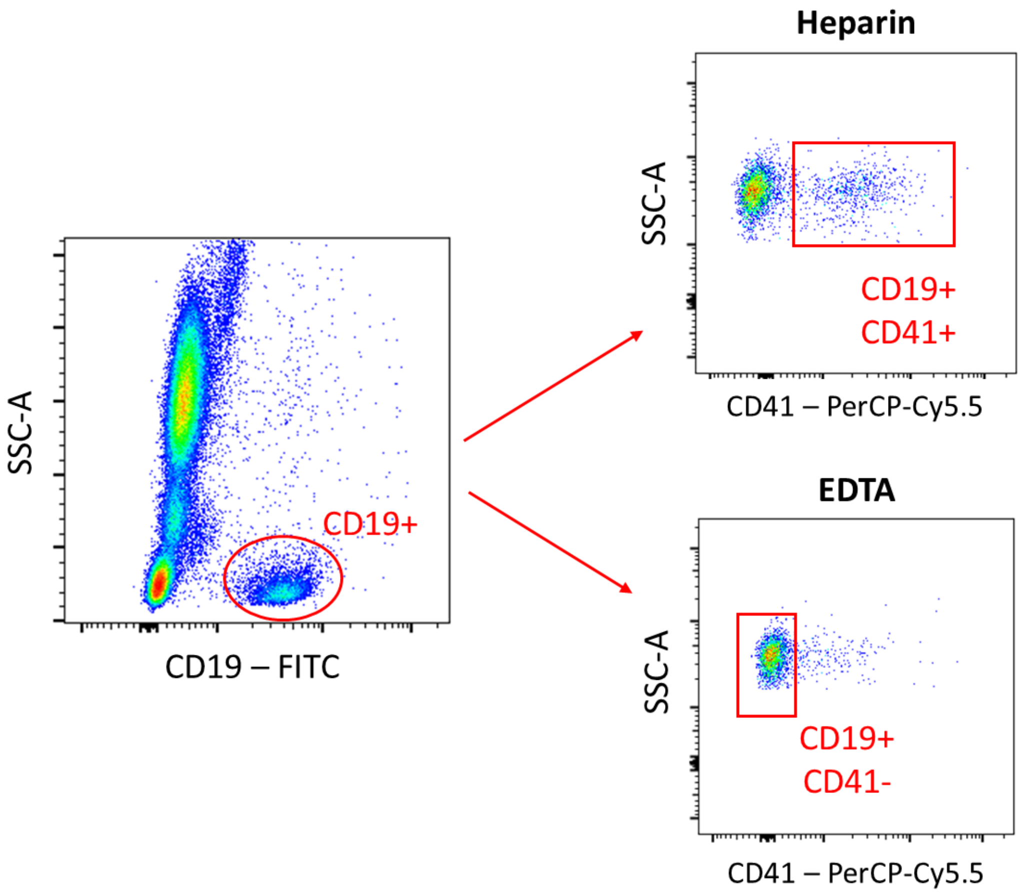

e-Figure 6. **Gating strategy for human B-lymphocyte detection in whole blood by flow cytometry.** B-lymphocytes were selected as a $CD19^+$ population and with a low SSC-A. In heparinized blood, B-lymphocyte-platelet complexes were selected as the $CD19^+CD41^+$ population, whereas platelet-free B-lymphocytes were gated as $CD19^+CD41^-$ from blood incubated with EDTA.

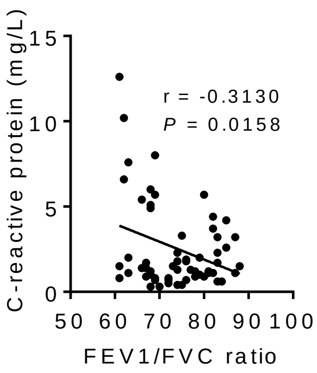

e-Figure 7. Circulating levels of C-reactive protein correlate negatively with FEV1/FVC ratio in these subjects. A negative correlation was found between FEV1/FVC ratio and circulating levels of C-reactive protein (mg/L). FEV1, forced expiratory volume in the first second; FVC, forced vital capacity.

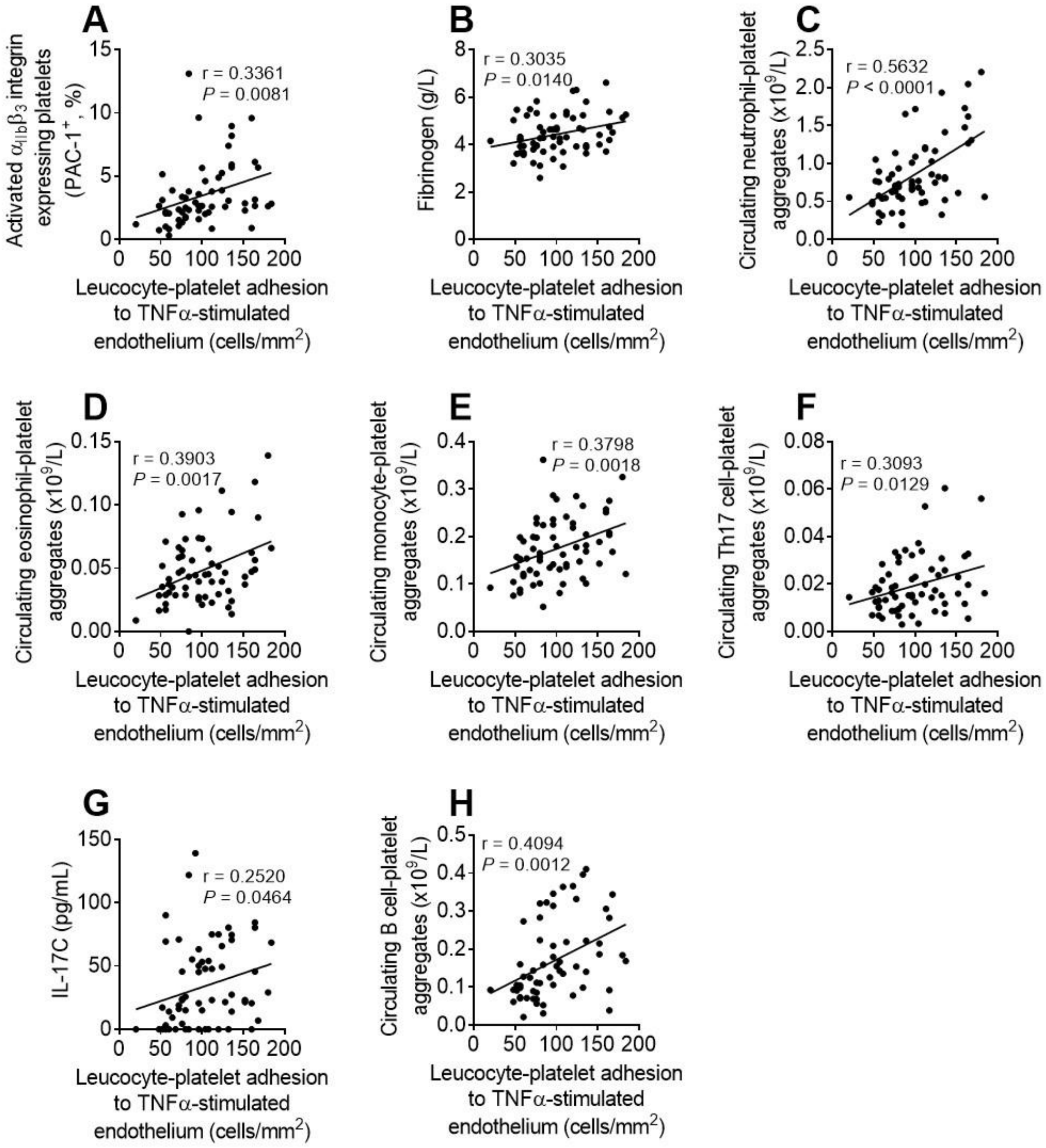

e-Figure 8. Platelet hyperactivity and IL-17C circulating levels correlate positively with leucocyte-platelet aggregate adhesiveness in these subjects. Positive correlations were found between leucocyte-platelet-endothelium interactions and several cellular and soluble parameters: platelet activation (**A**, percentage of PAC-1$^+$ platelets); absolute count ($\times 10^9$/L) of circulating neutrophil- (**C**), eosinophil- (**D**), monocyte- (**E**), Th17 cell- (**F**) and B cell-platelet aggregates (**H**); circulating levels of fibrinogen (**B**, g/L) and interleukin (IL)-17C (**G**, pg/mL).

References

1. Vestbo, J.; Edwards, L.D.; Scanlon, P.D.; Yates, J.C.; Agusti, A.; Bakke, P.; et al. Changes in forced expiratory volume in 1 second over time in COPD. *N Engl J Med*. 2011;*365*(13):1184-92. 10.1056/NEJMoa1105482

2. Harvey, B.G.; Strulovici-Barel, Y.; Kaner, R.J.; Sanders, A.; Vincent, T.L.; Mezey, J.G.; et al. Risk of COPD with obstruction in active smokers with normal spirometry and reduced diffusion capacity. *Eur Respir J*. 2015;*46*(6):1589-97. 10.1183/13993003.02377-2014

3. Global Initiative for Chronic Obstructive Lung Disease (GOLD). Global Strategy for Prevention, Diagnosis and Management of COPD: 2024 Report: GOLD; 2024 [accessed July 22nd, 2024]. Available from: https://goldcopd.org/wp-content/uploads/2024/02/GOLD-2024_v1.2-11Jan24_WMV.pdf.

4. Graham, B.L.; Steenbruggen, I.; Miller, M.R.; Barjaktarevic, I.Z.; Cooper, B.G.; Hall, G.L.; et al. Standardization of Spirometry 2019 Update. An Official American Thoracic Society and European Respiratory Society Technical Statement. *Am J Respir Crit Care Med*. 2019;*200*(8):e70-e88. 10.1164/rccm.201908-1590ST

5. Paliogiannis, P.; Fois, A.G.; Sotgia, S.; Mangoni, A.A.; Zinellu, E.; Pirina, P.; et al. Neutrophil to lymphocyte ratio and clinical outcomes in COPD: recent evidence and future perspectives. *Eur Respir Rev*. 2018;*27*(147):170113. 10.1183/16000617.0113-2017

6. Lourenço, J.D.; Ito, J.T.; Martins, M.A.; Tibério, I.; Lopes, F. Th17/Treg Imbalance in Chronic Obstructive Pulmonary Disease: Clinical and Experimental Evidence. *Front Immunol*. 2021;*12*:804919. 10.3389/fimmu.2021.804919

7. Postea, O.; Vasina, E.M.; Cauwenberghs, S.; Projahn, D.; Liehn, E.A.; Lievens, D.; et al. Contribution of platelet CX(3)CR1 to platelet-monocyte complex formation and vascular recruitment during hyperlipidemia. *Arterioscler Thromb Vasc Biol*. 2012;*32*(5):1186-93. 10.1161/atvbaha.111.243485

8. Marques, P.; Domingo, E.; Rubio, A.; Martinez-Hervás, S.; Ascaso, J.F.; Piqueras, L.; et al. Beneficial effects of PCSK9 inhibition with alirocumab in familial hypercholesterolemia involve modulation of new immune players. *Biomed Pharmacother*. 2022;*145*:112460. 10.1016/j.biopha.2021.112460
